# Depression symptoms are associated with demographic characteristics, nutritional status, and social support among young adults in Chile: a latent class analysis

**DOI:** 10.1186/s12889-024-20173-w

**Published:** 2024-10-11

**Authors:** Francisca Carvajal, José Manuel Lerma-Cabrera, Pía Herrera-Ponce de León, Sandra López-Arana

**Affiliations:** 1 Department of Psychology, Faculty of Psychology, University of Almeria, Almeria, Spain; 2 Health Research Center CEINSA, University of Almeria, Almeria, Spain; 3 Family Health Center Olmué, Olmué, Valparaíso, Chile; 4 Department of Nutrition, Faculty of Medicine, University of Chile, Av. Independencia 1027, Independencia, Santiago, Chile; 5 School of Nutrition and Dietetics, Faculty of Medicine, Finis Terrae University, Santiago, Chile

**Keywords:** Depression, Symptoms profile, Latent class analysis, Suicidal ideation, Young adult

## Abstract

**Background:**

Depressive disorders are a critical public health concern in Chile. Nonetheless, there is a lack of evidence regarding the identification of depressive symptom clusters. The objective was to identify depressive symptom clusters among Chilean young adults and examine how demographic, and lifestyle factors as well as social support can influence and predict them.

**Methods:**

Cross-sectional study conducted among 1,000 participants from the Limache cohort 2. A latent class analysis (LCA) was performed to identify depressive symptom clusters, using the Patient Health Questionnaire (PHQ-9). Multinomial logistic regression was then applied to explore the associations between identified classes and potential predictors. The models were adjusted by age and sex.

**Results:**

Three latent classes of depressive symptoms were identified: minimal (25.7%); somatic (50.7%) and severe (23.6%). In the severe class for eight out nine depressive symptoms the probabilities were above 50%, and the probability of suicidal ideation was almost a third in this class. Being female (Adjusted Odds ratio [AOR], 2.49; 95% confidence interval [CI] [1.63–3.81]), current smoker (AOR, 1.74; 95% CI [1.15–2.65]), having basic education (AOR, 3.12; 95% CI [1.30–7.53]) and obesity (AOR, 2.72; 95% CI [1.61–4.59]) significantly increased the likelihood of belonging to severe class. Higher social support decreased the odds of being in the somatic (OR, 0.96; 95% CI [0.93–0.98]) and severe (OR, 0.92; 95% CI [0.90–0.94]) classes.

**Conclusions:**

These findings highlight the importance of individualized intervention strategies for depression management. Also, the study suggests that nutritional status and social support should be considered when addressing depression in this population.

## Background

Depressive disorders have a significant global impact, affecting approximately 300 million people worldwide, including in Chile [1, 2]. The burden of mental disorders is led by depression, and it is the second of the top 25 causes of years lived with disability (YLD) [2, 3]. In Chile, approximately 22% of adults and children have experienced a mental disorder [4, 5], and annual prevalence of depressive disorder was 6.2% based on the Chilean National Health Survey 2016–2017 (ENS-2016-2017) [6].

Depression is a heterogeneous disorder characterized by persistent feelings of sadness and loss of interest or pleasure in usual life activities [7]. Its diagnosed is based on a cluster of signs and symptoms outlined in the Diagnostic and Statistical Manual of Mental Disorders, 5th edition (DSM-5) [8]. Depressive symptoms can be classified into three dimensions: (1) “core symptoms” that often includes depressed mood or anhedonia, (2) “cognitive symptoms,” such as impaired ability to think or concentrate, as well as feelings of worthlessness, guilt, and suicidal thoughts and (3) “somatic symptoms,” such as sleep problems, fatigue or loss of energy, and changes in appetite or psychomotor agitation/retardation [9, 10].

Previous studies on depression often use total scores on scales as screening tool [11–14], but this approach may overlook specific patterns of symptoms and their clinical implications in the treatment [15, 16]. To address this limitation, data-driven approaches like latent class analysis (LCA) have gain popularity [17, 18]. However, much of the evidence from LCA in depressive disorders research comes from high-income countries, psychiatric hospitals, outpatient clinics and primary care settings [16, 19, 20], limiting its generalization [21]. Additional information is needed to further document the extent of depressive symptom clusters in the general population and in low and middle-income countries.

Depression is multifactorial and has been related to certain biological and environmental factors, including age, gender, nutritional status, ethnicity, as well as socioeconomic status [22–25]. However, there is limited research on how these variables impact on the symptom profile of depression [24, 26]. To address this gap, we conducted a study using LCA to identify depressive symptoms clusters and examine the differences in sociodemographic characteristics, lifestyle, nutritional status and social support among young adults enrolled in the Limache Cohort 2, one of the longest birth cohort studies in Latin America [27–29]. By using LCA in a diverse cohort, we aim to gain a better understanding of depressive symptoms cluster in the general population, particularly in countries such as Chile that has undergone a significant economic transition from a low-and-middle-high-income country to a high-income country over the past two decades. Previous evidence from Chile has reported that the prevalence of depression has been sustained over time [30, 31], but varies by regions [32], and particularly impacts women [33], individuals with lower levels of education [34], economically disadvantage [35, 36] and with lower quality of the built environment [37]. This research can provide important insights into the heterogeneity of depression and inform individualized assessment and treatment approaches.

## Methods

### Data and sample

This study is cross-sectional and utilizes information from the Limache cohort 2. The study design has been described elsewhere [28, 29, 38, 39]. In summary, a random sample of 1000 subjects from a sampling frame of 3848 singleton livebirths that occurred between 1988 and 1992 at the Limache hospital [39] was assessed between 2014 and 2018. The hospital´s catchment area encompasses the municipalities of Limache and Olmué. In Limache, 84.9% and 15.1% of participants reside in urban and rural areas respectively. In Olmué, 70.1% live in urban areas, with 29.9% in rural areas [40].

## Measures

Participants completed a sociodemographic questionnaire that included information on age, sex, education level categorized as (i) basic or incomplete secondary; (ii) complete secondary and (iii) higher. Socioeconomic status was determined using the methodology of the World Association of Market Research, as recommended by ADIMARK (the main market research and public opinion organization in Chile) [41]. A matrix based on education level and occupation of head of the household provides five socioeconomic categories: ABC1 (high); C2 (upper-middle); C3 (middle); D (lower-middle); and E (low).

Trained personnel measured participants´ weight and height using standardized procedures [42] to calculate body mass index (BMI) categories as: (i) underweight (BMI < 18.5 kg/m^2^); (ii) normal (BMI 18.5–24.9 kg/m^2^); (iii) overweight (25.0–29.9 kg/m^2^) and (iv) obesity (≥ 30 kg/m^2^).

Physical activity level was assessed using the International Physical Activity Questionnaire (IPAQ) [43] and classified as (i) low (< 599 (Metabolic Equivalents-minutes/week) (MET-min/week)); (ii) moderate (600–3000 MET-min/week) and (iii) high (> 3000 MET-min/week). Smoking status was classified as current or nor smoking. The Alcohol Use Disorders Identification Test (AUDIT) was used to identify hazardous and harmful alcohol consumption [44]. Scores of 0 to 7 were considered low risk, 8 to 15 as moderate risk, and higher than 16 as high risk and alcohol dependence.

Social support was measured using the Medical Outcomes Study-Social Support Survey (MOS-SSS) [45], a 19-item self-report instrument that assesses four dimensions: emotional/informational support, tangible support, affectionate support and positive social interaction. Responses ranged from 1 *(none of the time)* to 5 *(all of the time)* and were transformed from 0 to 100 for each subscale, with higher values indicating higher levels of care and social support. The Chilean version of MOS-SSS has shown good reliability and validity [46].

Depression was assessed using the Patient Health Questionnaire (PHQ-9), which has a good validity and reliability in the Chilean setting [47]. The PHQ-9 consists of nine items scored on a Likert scale ranging from 0 (“not at all”) to 3 (“nearly every day”). Scores of 10–14 were considered moderate depression, between 15 and 19 moderately severe depression, and a score of 20 to 27 severe depression [48]. The nine items of PHQ-9 were dichotomized as present or absent to perform the Latent Class Analysis (LCA).

### Statistical analysis

All analyses were conducted in Stata statistical software version 17.0 (StataCorp LLC). We assessed the normal distribution of continuous variables using Shapiro Wilk test. Non-normal variables were reported as medians (25th and 75th percentiles), while categorical variables were presented as absolute frequencies and percentages. Categorical and continuous variables were compared by sex using chi-square test and Mann-Whitney U test.

LCA was conducted five times using one to five classes to identify the best-fitting model for the data. LCA is a probabilistic clustering approach that describes the distribution of data and evaluates the probability of respondents belonging to certain latent classes [17, 18]. A combination of the statistical indicators, including the Bayesian information criterion (BIC), the Akaike information criterion (AIC), and the likelihood ratio for goodness-of-fit statistic (G^2^), was used to decide on the best-fitting model, considering the interpretability of the models.

To determine a trend between sociodemographic, lifestyle and social support characteristics and the LCA classes identified we performed Jonckheere-Terpstra test. In addition, we examined their relationship using multinomial logistic regression model. Firstly, we performed unadjusted analysis for each possible predictor variable. Secondly, we adjusted our models for sex and age. Interactions were tested to examine whether the association of predictors differed by sex and age, but these were not statistically significant. We therefore did not stratify our analysis.

## Results

The median age of the study population was 24.9 years (interquartile range [IQR] = 23.8–26.4), with 56.9% females. Only 6.5% had completed primary education or less. There were significant differences between sexes in the educational level, with women having a lower proportion of individuals with a low educational level and a higher proportion with more than 13 years of education than men. Most participants belonged to the medium socioeconomic status, with only 4% in the high category. The median BMI was 26.4 kg/m^2^, and there were no differences in BMI between sexes. In addition, females were more likely to have lower rates of intense physical activity and smoking compared to males. There were also differences by sex in the tangible and affectionate social support dimensions. The median score and percentiles were lower in women than in men in the tangible support dimension. In contrast, men exhibited lower scores than women in the dimension of affectionate support (Table 1).

**Table 1:** Demographic, lifestyle and social support characteristics of the sample among young adults from Limache cohort 2 by sex

Characteristic	Total	Male	Female	*P*-value
	*n* = 1000	*n* = 431	*n* = 569	
**Educational level (%)**				
Basic (< 8 y)	6.5	8.8	4.8	
Medium (9–12 y)	45.4	47.1	44.1	**0.01** ^**1**^
High (≥ 13 y)	48.1	44.1	51.1	
**Socioeconomic status (%)**				
Low	14.1	14.6	13.7	
Medium low	42	45.9	39	
Medium	27.1	23	30.2	0.09^1^
Medium High	12.4	11.8	12.8	
High	4.4	4.6	4.2	
**Physical activity (%)**				
Low (< 600 METs min/w)	38.5	28.1	46.4	**0.01** ^**1**^
Moderate (600–3000)	34.6	34.6	34.6	
Intense (> 3000)	26.9	37.4	19	
**BMI Median** (P_25_–P_75_)	26.4 (23.4–30.7)	26.4 (23.7–29.9)	26.3 (23.3–31.0)	0.73^2^
**Nutritional status (%)**				
Underweight (< 18.5)	1.6	2.1	1.2	
Normal (18.5–24.9)	36.4	34.6	37.8	0.09^1^
Overweight (25-29.9)	35	38.8	32.2	
Obesity (≥ 30)	27	24.6	28.8	
**Alcohol intake (%)** ^**a**^				
Low risk (0–7)	89.5	90	89.2	0.92^1^
Moderate risk (8–15)	8.7	8.3	8.9	
High Risk/Alcohol dependence (> 16)	1.8	1.8	1.9	
**Smoking (%)**				
Yes	39.6	43.6	36.6	**0.03** ^**1**^
No	60.4	56.4	63.4	
**MOS-SSS Median (P**_**25**_ **–P**_**75)**_				
Emotional/information support	90.0 (75.0–100)	90.0 (75.0–100)	92.5 (75.0–100)	0.57^2^
Tangible support	90.0 (70.0–100)	95.0 (75.0–100)	95.0 (75.0–100)	0.003^2^
Affectionate support	100 (86.7–100)	100 (86.7–100)	100 (93.3–100)	0.01^2^
Positive social interaction	95.0 (80.0–100)	95.0 (80.0–100)	90.0 (75.0–100)	0.06^2^
Overall support	86.0 (73.5–94.0)	86.0 (74.0–94.0)	86.0 (73.0–94.0)	0.39^2^

^1^Pearson’s Chi square

^2^Mann-Whitney U test

^a^*n* = 925

The three-class model was found to be the best solution in terms of interpretability, and models four and five were discarded to avoid convergence failures (Table 2).

**Table 2:** Comparison of LCA models with different latent class based on model selection statistics

Latent class	Goodness of fit			Ratio to belong to a latent class (%)				
	AIC	BIC	G^2^	1st	2nd	3rd	4th	5th
**1**	10323.65	10367.82	1521.21	100.0				
**2**	9491.15	9584.39	668.70	71.2	28.8			
**3**	**9337.39**	**9479.72**	**494.95**	**25.7**	**50.7**	**23.6**		
**4**	9320.95	9512.36	458.51	31.1	39.3	6.8	22.8	
**5**	9282.12	9522.60	399.68	30.5	6.7	39.5	19.7	3.7

**AIC**: Akaike information criterion

**BIC**: Bayesian information criterion

**G**^**2**^: Likelihood ratio for goodness-of-fit statistic

**Fig. 1:**
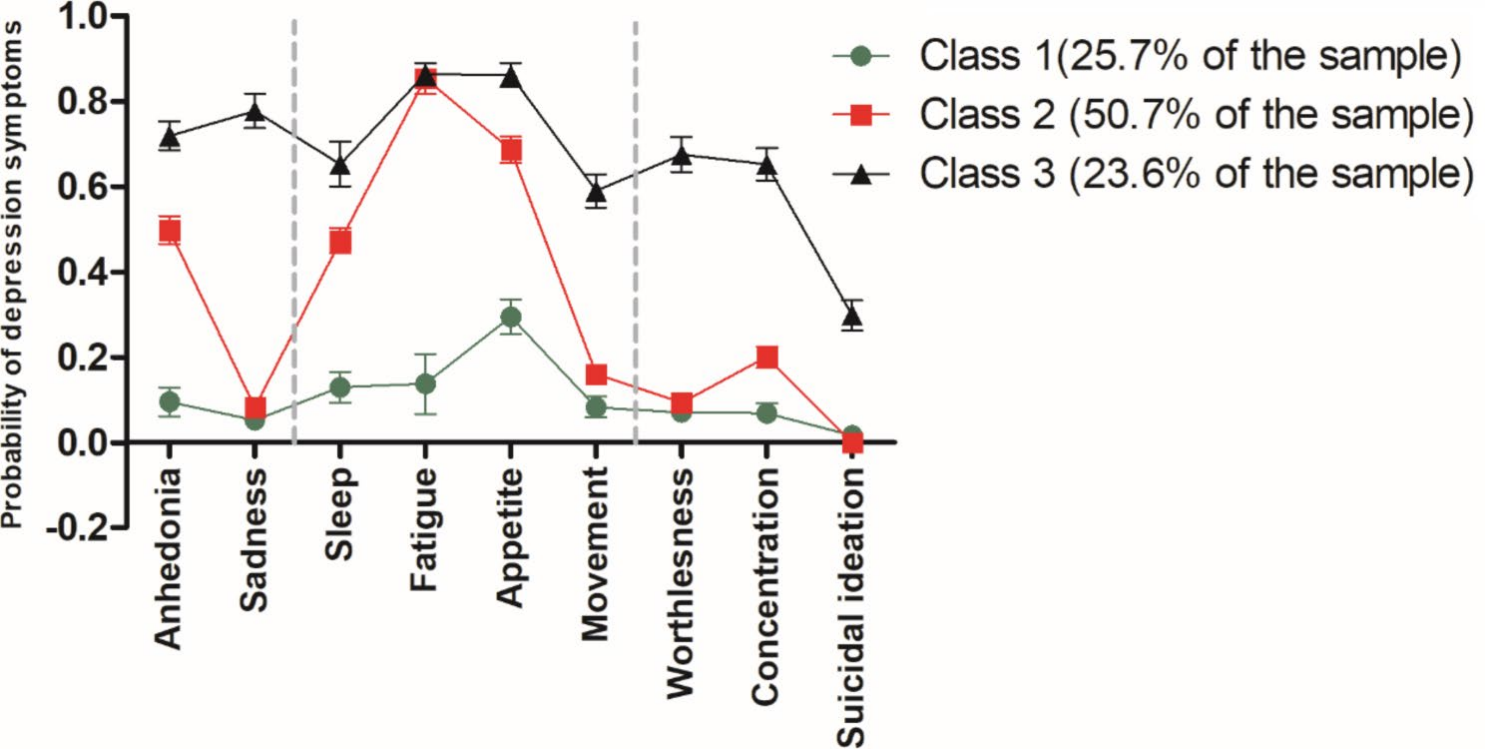
The three-class model and probabilities of nine depressive symptoms among young adults in the Limache cohort 2

Based on PHQ-9 scores, 13.2% of participants were considered as moderately severe and 6.0% as severe. Figure 1 shows the probability of nine depressive symptoms within each class for the three-class model. Class 1 (25.7% of participants) was labeled the “minimal symptoms” class, showing the lowest score probabilities for most depressive symptoms, except for appetite disturbances (29.4%). The class 2 (50.7% of participants) was defined as the “somatic symptoms’’ class, with high probabilities of fatigue (85.3%), appetite disturbances (68.7%) and sleep problems (47.3%), as well as anhedonia (49.7%). Finally, class 3 (23.6%) was labeled as “severe symptoms’’ class, with probabilities above 50% for eight out of nine depressive symptoms. Notably, this class exhibited a higher occurrence of fatigue (86.3%), appetite changes (86.0%), followed by sadness (77.7%), anhedonia (71,9%), worthlessness (67.5%) and sleep problems (65.2%). Additionally, the probability of suicidal ideation was approximately one-third in this class.

Statistically significant differences in sex, educational level, lifestyle, and social support characteristics were found between the three-class models, except for socioeconomic status, and alcohol intake. Overall, women, participants with lower education, a higher obesity rate, and a higher BMI, smokers, and those with less social support, were more likely to belong to the “severe symptoms” class (Table 3).

**Table 3:** Summary statistics of the three-class models among young adults from Limache cohort 2

Characteristic	Minimal symptoms	Somatic symptoms	Severe symptoms	*P*-value
	*n* = 243	*n* = 526	*n* = 231	
**Sex (%)**				
Male	52.7	44.3	30.3	**< 0.001** ^**1**^
Female	47.3	55.7	69.7	
**Educational level (%)**				
Basic (< 8 y)	4.9	6.3	8.7	
Medium (9–12 y)	39.9	44.3	53.7	**0.001** ^**1**^
High (≥ 13 y)	55.1	49.4	37.7	
**Socioeconomic status (%)**				
Low	12.3	14.3	15.6	
Medium low	37.9	42.4	45.5	
Medium	35	24.1	25.5	0.10^1^
Medium High	11.5	14.1	9.5	
High	3.3	5.1	3.9	
**Physical activity (%)**				
Low (< 600 METs min/w)	42.4	33.8	45	0.72^1^
Moderate (600–3000)	38.3	32.5	35.5	
Intense (> 3000)	19.3	33.7	19.5	
**BMI** Median (P_25_–P_75_)	25.5 (22.9–28.8)	26.3 (23.5–30.4)	27.5 (24.4–32.0)	**< 0.001** ^**2**^
**Nutritional status (%)**				
Underweight (< 18.5)	1.2	1.7	1.7	
Normal (18.5–24.9)	44	35.9	29.4	**0.002** ^**1**^
Overweight (25-29.9)	35.8	35.6	32.9	
Obesity (≥ 30)	18.9	26.8	35.9	
**Alcohol intake (%)** ^**a**^				
Low-risk (0–7)	89.5	88.7	91.5	0.17^1^
Moderate risk (8–15)	8.8	9.5	6.6	
High Risk/Alcohol dependence (> 16)	1.8	1.9	1.9	
**Smoking (%)**				
Yes	36.2	38.4	45.9	10.01^1^
No	63.8	61.6	54.1	
**MOS-SSS Median (P**_**25**_ **–P**_**75**_ **)**				
Emotional/information support	97.5 (82.5–100)	92.5 (77.5–100)	80.0 (57.5–95.0)	**< 0.001** ^**2**^
Tangible support	95.0 (85.0–100)	90.0 (75.0–100)	80.0 (60.0–95.0)	**< 0.001** ^**2**^
Affectionate support	100 (93.3–100)	100 (93.3–100)	93.3 (73.3–100)	**< 0.001** ^**2**^
Positive social interaction	100 (85.0–100)	95.0 (80.0–100)	80.0 (65.0–95.0)	**< 0.001** ^**2**^
Overall support	91.0 (81.0–95.0)	87.0 (75.0–94.0)	76.0 (63.0–89.0)	**< 0.001** ^**2**^

^1^P-trend from Jonckheere-Terpstra test

^2^Kruskal-Wallis test

^a^*n* = 925

Associations between sex, educational level, socioeconomic status, nutritional status, lifestyle indicators as well as social support and clusters of depression symptoms subgroups are shown in Table 4, with the “minimal symptoms” class as the reference group. Having low or moderate physical activity significantly decreased the odds of being in the “somatic symptoms” class. Similarly, having higher scores of emotional/information, tangible, and overall support were associated with a decreased likelihood of belonging to the “somatic symptoms” class. While the probability of being in the “somatic symptoms” class was higher among individuals with obesity.

**Table 4:** Predictors of membership in latent classes of depressive symptoms among young adults in the Limache cohort 2

	Somatic symptoms class		Somatic symptoms class		Severe symptoms class		Severe symptoms class	
Predictors	Unadjusted		Adjusted		Unadjusted		Adjusted	
	OR (95% CI)	*p*-value	OR (95% CI)	*p*-value	OR (95% CI)	*p*-value	OR (95% CI)	*p*-value
**Sex**								
Female	1.42 (0.96–2.10)	0.08	1.43 (0.97–2.12)	0.07	**2.49 (1.62–3.81)**	**< 0.001**	**2.49 (1.63–3.81)**	**< 0.001**
Male	Reference		Reference		Reference		Reference	
**Educational level**								
Basic	1.14 (0.48–2.74)	0.76	1.33 (0.55–3.27)	0.76	**2.35 (1.02–5.45)**	**0.04**	**3.12 (1.30–7.53)**	**0.01**
Medium	1.25 (0.83–1.88)	0.29	1.26 (0.83–1.91)	0.28	**2.19 (1.43–3.36)**	**< 0.001**	**2.39 (1.54–3.71)**	**< 0.001**
High	Reference		Reference		Reference		Reference	
**Socioeconomic status**								
Low	0.59 (0.17–2.00)	0.40	0.58 (0.17–1.97)	0.38	100 (0.28–354)	1.00	1.03 (0.28–3.72)	0.97
Medium low	0.62 (0.20–1.95)	0.41	0.59 (0.18–1.86)	0.36	1.02 (0.31–3.33)	0.98	1.05 (0.31–3.51)	0.94
Medium	0.37 (0.12–1.17)	0.09	0.34 (0.11–1.07)	0.07	0.52 (0.16–1.74)	0.29	0.49 (0.14–1.67)	0.25
Medium high	0.67 (0.19–2.28)	0.51	0.64 (0.19–2.22)	0.48	0.59 (0.16–2.17)	0.43	0.57 (0.15–2.17)	0.41
High	Reference		Reference		Reference		Reference	
**Physical activity**								
Low	**0.28 (0.17–0.46)**	**< 0.001**	**0.23 (0.13–0.39)**	**< 0.001**	0.91 (0.52–1.58)	0.73	0.69 (0.39–1.25)	0.22
Moderate	**0.32 (0.19–0.54)**	**< 0.001**	**0.29 (0.17–0.49)**	**< 0.001**	0.80 (0.46–1.42)	0.45	0.69 (0.38–1.23)	0.21
Intense	Reference		Reference		Reference		Reference	
**BMI**	1.02 (0.98–1.06)	0.31	1.02 (0.98–1.06)	0.31	**1.05 (1.01–1.08)**	**0.02**	**1.04 (1.01–1.08)**	**0.02**
**Nutritional status**								
Underweight	1.95 (0.36–10.6)	0.44	2.06 (0.40–10.5)	0.38	2.76 (0.51–15.0)	0.24	3.08 (0.59–17.1)	0.18
Normal	Reference		Reference		Reference		Reference	
Overweight	1.14 (0.72–1.79)	0.58	1.21 (0.76–1.92)	0.41	1.38 (0.86–2.20)	0.18	1.52 (0.94–2.45)	0.09
Obesity	1.69 (0.99–2.87)	0.05	**1.76 (1.03–3.00)**	**0.04**	**2.61 (1.56–4.37)**	**< 0.001**	**2.72 (1.61–4.59)**	**< 0.001**
**Alcohol intake**								
Low risk (0–7)	Reference		Reference		Reference		Reference	
Moderate risk (8–15)	1.16 (0.57–2.37)	0.68	1.17 (0.57–2.40)	0.67	0.60 (0.26–1.40)	0.24	0.59 (0.25–1.39)	0.22
High risk/Alcohol dependence (> 16)	1.58 (0.36–6.92)	0.54	1.55 (0.35–6.88)	0.57	0.91 (0.15–5.44)	0.92	0.97 (0.16–5.85)	0.97
**Smoking**								
Yes	1.14 (0.73–1.76)	0.57	1.22 (0.79–1.90)	0.37	**1.74 (1.15–2.65)**	**0.009**	**1.93 (1.25–2.96)**	**0.003**
No	Reference		Reference		Reference		Reference	
**MOS-SSS**								
Emotional/information support	**0.98 (0.96–0.99)**	**< 0.005**	**0.98 (0.96–0.99)**	**0.003**	**0.95 (0.93–0.97)**	**< 0.001**	**0.94 (0.93–0.96)**	**< 0.001**
Tangible support	**0.97 (0.95–0.98)**	**< 0.001**	**0.97 (0.95–0.98)**	**< 0.001**	**0.95 (0.94–0.97)**	**< 0.001**	**0.95 (0.94–0.97)**	**< 0.001**
Affectionate support	0.99 (0.97–1.01)	0.29	0.99 (0.96–1.01)	0.25	**0.95 (0.94–0.97)**	**< 0.001**	**0.95 (0.93–0.97)**	**< 0.001**
Positive social interaction	0.98 (0.97–1.00)	0.1	0.98 (0.97–1.00)	0.11	**0.95 (0.93–0.96)**	**< 0.001**	**0.95 (0.93–0.96)**	**< 0.001**
Overall support	**0.96 (0.94–0.98)**	**<0.001**	**0.96 (0.93–0.98)**	**<0.001**	**0.92 (0.90–0.94)**	**<0.001**	**0.92 (0.90–0.94)**	**<0.001**

OR = Odds ratio, 95% CI = Confidence interval

The reference category is minimal symptoms class

Models were adjusted by sex and age

The probability of being in the “severe symptoms” class was higher among females, those with basic and medium educational levels, those with a higher BMI, obesity as their nutritional status and being current smokers. In contrast, having higher scores on the four dimensions of social support, as well as the overall score, decreased the odds of membership in the “severe symptoms” class in comparison to the “minimal symptoms” class.

## Discussion

Using LCA, we identified three clusters of depressive symptoms among young adults from the Limache cohort 2. The findings show phenotypic heterogeneity in the manifestation of depressive symptomatology in our participants, with three-quarters falling under somatic (50.7%) and severe (23.6%) classes. Depression is typically assessed using a validated scale that provides a summary score indicating symptom severity. However, like in our study this condition is highly heterogenous and the symptoms can differ significantly [15]. The prevalence of depressive disorder in our sample was higher than rates reported in the last ENS-2016-2017 [6]. This discrepancy could be explained based on the instruments itself. PHQ-9 has been specifically designed as a screening tool for current depressive symptoms experienced over the past two weeks, while the Composite International Diagnostic Interview (CIDI) is a well-known tool used to assess clinical diagnosis of major depression during the past twelve months [47, 48]. In addition, PHQ-9 is less strict than CIDI to estimate the temporal pattern for symptoms occurrence. It is likely to encompass as cases people with symptoms that were possible less severe.

To our knowledge, this is the first study to use LCA to identify cluster of depressive symptoms using PHQ-9 in a sample of non-institutionalized Chilean young adults that usually do not attend primary health care or outpatients´ facilities. The reason for the low attendance could be explained by the low interest of Chilean young adults in health promotion and disease prevention. In 2018, just 13% of women and 16% of men aged 20 to 65 years used preventive services in the country. Similar figures were observed among people from Limache and Olmué (https://reportesrem.minsal.cl/). Previous research in Chile using LCA was limited to a convenience sample of 297 depressed patients with a more severe and complex diagnosis than in our research, mostly women from the Chilean Primary Health Care, using different screening and diagnostic tools for depressive disorders, Mini International Neuropsychiatric Interview and the Hamilton Depression Rating Scale [49].

Our results align with others international studies that identified three to five clusters of depressive symptoms using PHQ-9 for depression screening [50–54]. Despite variations in the number of classes and individuals, LCA remains a valuable analytical tool for studying the heterogeneity of mental disorders. It considers maximum likelihood for subgroups, ensuring internally homogeneous and externally heterogeneous estimations. LCA also provides fit statistics and probabilities within each class, aiding in selecting the most appropriate model [55, 56].

In the current study, application of a data-driven approach yielded that the three-class model (i) minimal symptoms, ii) somatic symptoms and iii) severe symptoms) presented the best statistical solution. The “somatic symptoms” class, the largest subtype, showed a low probability of cognitive symptoms, but a significantly high proportion of fatigue, appetite disturbances and sleep problems with a slightly higher anhedonia than the “minimal symptoms” class. This class aligns with the evidence that suggest two-thirds of depressed patients initially present only somatic symptoms [57].

Somatic symptoms have often been used as indicators of latent classes of depressive symptoms in individuals with major depressive disorder [58], but they can also be non-specific to depressive disorders and be present in other diagnoses, such as Generalized Anxiety Disorder (GAD). Network models have demonstrated that somatic symptoms can act as a link (or nodes) connecting symptoms of other mental disorders [59, 60]. It is likely that sleeping problems may lead to fatigue and lack of concentration, which can trigger mood-related symptoms and anhedonia. It is not clear whether somatic symptoms are the cause or consequence of depressive disorders, and further investigation is needed to explain this pattern. Recent studies have suggested that somatic symptoms play a crucial role in the trajectories of depression [61–63]. It is a well-documented phenomenon that somatic symptoms are very common in depressive disorders across all cultures and care settings [64–66]. In our study, we observed that the severe symptoms class grouped individuals who in addition to core symptoms, also exhibited elevated somatic symptoms. This finding is in line with previous research showing that more severe or numerous somatic symptoms are consistently associated with increased depression severity, and lower remission rates [64].

To better understand the underlying neurobiology of depression, there are associations between somatic symptoms and inflammation. It has been shown that patients with major depressive disorders had increased inflammatory markers such as interleukin (IL)-1β, IL-6, tumor necrosis factor (TNF)-α and C-reactive protein (CRP) [7, 9]. Recent studies have also shown elevated levels of other biomarkers involved in the sleep and appetite regulation such as ghrelin, orexin and nesfatin-1, in depressed patients [7, 67]. Consistently, depressed patients with increased appetite/weight often carry a higher number of genetic risk variants for high body mass index (BMI) and CRP [68]. Higher BMI has been proposed as a linking pathway between somatic symptoms of depression and elevated inflammation [9]. Obesity, characterized by excessive accumulation of adipose tissue leading to chronic low-grade inflammation, leptin and insulin resistance, and altered activity of the hypothalamic-pituitary-adrenal (HPA) axis, potentially triggering depressive symptoms directly or indirectly [69]. Peripheral inflammation may lead to the stimulation of activate the vagus nerve, which subsequently stimulates an inflammatory response in neural circuits associated with depression [68–70]. These findings highlight the complex relationship between somatic symptoms, inflammation, and depression, pointing to potential avenues for understanding and treating depressive disorders. Nonetheless, we were unable to establish a definitive causal relationship due to the cross-sectional nature of our study.

In our study, we observed that individuals in the somatic and severe symptoms classes had higher median BMI and greater prevalence of obesity compared to those in the minimal symptoms class. The prevalence of obesity has been increasing in Chile in recent years [6]. However, there have been few studies examining the relationship between obesity and depressive symptoms in the country. To our knowledge, only Blümel and collaborators found that obesity was associated with depressive symptoms in middle-aged Chilean women [71]. A meta-analysis of longitudinal studies found a bidirectional association between obesity and depression. People with obesity faced 55% more risk to developing depression over time, while those with depression had a 58% increased risk of obesity [72]. As mentioned above, one possible explanation for this association is that obesity may lead to a pro-inflammatory state, which could exacerbate depressive symptoms [9]. However, it should not be forgotten that psychological and social mechanisms (e.g., body image dissatisfaction, stigmatization and self-concept) could also contribute to this pattern [73].

Demographic characteristics such as sex and educational level significantly predicted the probability of belonging to the class with severe symptoms. Women had 2.5 times more likelihood to belong to the severe class than men. These findings are consistent with existing research in Chile, which indicates that the prevalence of depressive symptoms varies by gender, being almost twice as high in women [6]. A meta-analysis found that females had higher levels of major depression and depression symptoms than males (OR = 1.95, 95% CI = 1.88, 2.03) [74]. A number of biological factors (genetic predisposition, hormonal or physiological stress response) and psychological factors (temperament or coping styles among others) may contribute to this gender gap; however, environmental factors such as societal structural gender inequality, appear to play a predominant role in this difference [22, 75].

Consistent with previous studies, lower education was associated with a greater risk of belonging to the class with more severe symptoms [76, 77]. Higher education often correlates with better occupational opportunities and income stability, which may provide individuals with greater access to social support [78]. Our study also supports the idea proposed by Adams et al., (2016) that higher level of social support decreased the odds of being in both somatic and severe classes [79]. Social support might act as protective role, buffering individuals against daily life stressors through access to tangible resources, emotional support, and informational support. The positive impacts of social support on physical and mental health have also been well-documented [79, 80]. Physical activity can also contribute to enhanced social support as it provides opportunities for interaction and socialization, which may improve self-management of depressive symptoms [81]. Furthermore, as previous studies have already shown, we found that being a current smoker significantly increased the likelihood of belonging to the severe class [82, 83]. Taken as a whole, these findings emphasize the importance of addressing social and lifestyle factor in depression management strategies.

The strengths of our study are related primarily to the Limache cohort itself, one of the longest birth cohort studies in Latin America. Therefore, our results allowed us to gain a better understanding of depressive symptom profile, which has been underexplored in this region compared to high-income countries. Additionally, we used a powerful data-driven approach like LCA to identify specific profiles of symptoms, ensuring that no crucial information about depression symptoms and their associations with other characteristics was overlooked. However, there are some limitations to consider. Our findings are specific to young adults aged 24–28 years in the Limache cohort and may not be representative of young adults at the national level. Comparisons with nationally representative surveys show a good agreement in sociodemographic variables such as educational level and sex, but differs in variables such as obesity, smoking and alcohol consumption [6, 84]. The cross-sectional nature of our analysis also restricts us from drawing causal inferences, due to this design is unable to establish temporal relationships and the possibility of reverse causality, since both the exposure and outcome are measured at the same point in time. Furthermore, residual confounding might influence the magnitude and direction of the associations between sociodemographic characteristics, lifestyle, nutritional status and social support and depressive symptoms clusters. Nonetheless, our adjusted regression models were used to minimize the impact of potential confounding factors, and the associations remained statistically significant and they were not deviated in their magnitude and direction. On the other hand, while LCA is a robust statistical method, it does have certain limitations. LCA categorizes individuals into classes based on the likelihood of their membership, inferred from their probabilities on indicator variables; however, this does not guarantee accurate class assignments [17]. To overcome this situation, we used the statistical indicators such as BIC, AIC and G^2^ to determine the optimal number of classes and labeled the classes based on the observable data trends trying to avoid the “naming fallacy” [56].

In conclusion, the identification of specific clusters of depressive symptoms can be a valuable in developing personalized intervention for depression management. We observed that these clusters were associated with specific biological and environmental characteristics, particularly among women, those with lower education, low social support, and higher body mass index or obesity. An understanding of these associations can assist in the prioritization of these aspects within the framework of depression disorder management strategies. Further research is needed to validate and expand on our findings to improve depression care in diverse populations.

## Data Availability

No datasets were generated or analysed during the current study.

## Abbreviations

LCA: Latent Class Analysis
PHQ-9: Patient Health Questionnaire
OR: Odds Ratio
BMI: Body Mass Index
IPAQ: International Physical Activity Questionnaire
AUDIT: Alcohol Use Disorders Identification Test
MOS-SSS: Medical Outcomes Study Social Support Survey
